# Endothelial Cell Contributions to COVID-19

**DOI:** 10.3390/pathogens9100785

**Published:** 2020-09-25

**Authors:** Alexandra E. Oxford, Fabio Halla, Evan B. Robertson, Brad E. Morrison

**Affiliations:** 1 Department of Biological Sciences, Boise State University, Boise, ID 83725, USA; aliceoxford@u.boisestate.edu (A.E.O.); fabiohalla@u.boisestate.edu (F.H.); evanrobertson2651@gmail.com (E.B.R.); 2 Biomolecular Ph.D. Program, Boise State University, Boise, ID 83725, USA

**Keywords:** COVID-19, coronavirus, SARS-CoV-2, endothelial, vascular, transdifferentiation, exosomes

## Abstract

Understanding of the clinical, histological and molecular features of the novel coronavirus 2019 (Severe Acute Respiratory Syndrome Coronavirus 2 (SARS-CoV-2)) has remained elusive. Coronavirus disease 2019 (COVID-19) caused by this virus has unusual clinical presentation with regard to other related coronaviruses. Recent reports suggest that SARS-CoV-2, unlike other related viruses, infects and replicates within endothelial cells, which may explain a significant portion of the observed clinical pathology. Likewise, mounting evidence associates vascular and endothelial cell dysfunction with increased mortality. This review focuses on understanding how endothelial cell pathology is caused by SARS-CoV-2 at the molecular and cellular levels and how these events relate to COVID-19. A detailed examination of current knowledge regarding canonical inflammatory reaction pathways as well as alteration of endothelial cell-derived exosomes and transdifferentiation by SARS-CoV-2 is included in this assessment. Additionally, given an understanding of endothelial contributions to COVID-19, potential therapeutic aims are discussed, particularly as would affect endothelial function and pathology.

## 1. Introduction

The new strain of coronavirus (Severe Acute Respiratory Syndrome Coronavirus 2 (SARS-CoV-2)) has created a global health crisis that is unlike any pandemic witnessed since the Spanish Flu in 1912. It is now reported that 40% of coronavirus disease-19 (COVID-19)-related deaths come from cardiovascular complications, with most of the remaining 60% attributed to respiratory failure separate from myocardial injury and unknown causes [1,2]. Many cases of SARS-CoV-2 infection are asymptomatic, causing subclinical or mild symptoms [3]. However, immunologic overdrive has been strikingly common in those who progressed to a later stage of coronavirus disease (COVID-19), presenting some similar clinical findings as those associated with macrophage activation syndrome (MAS) [4]. The associated increase in serum cytokines (termed a “cytokine storm”) progresses to cause acute respiratory distress syndrome (ARDS). In addition, endothelial cell infection promotes the formation of thrombus associated with an elevated neutrophil count [5]. It is important to note that though endothelial infection has been validated, the virus remains highly replicative in the throat and upper respiratory tract (as is symptomatically consistent with respiratory viruses of this type), and has been found to be inactive (un-replicative) within the blood [6]. While the cardiovascular complications in patients with COVID-19 are becoming more apparent, the underlying mechanisms leading to these issues are not fully understood. This review will focus on the concept of endothelial cell infection and dysfunction as an active driver of COVID-19, which begins as a respiratory illness, with vascular pathology contributing significantly to the most negative patient outcomes.

## 2. Progression and Pathology of COVID-19

To better explain the varying symptoms and severities that arise from SARS-CoV-2 infection, the stepwise progression of COVID-19 is utilized. Disease progression consists of three phases: the early infection phase, the pulmonary phase, and the hyperinflammation phase [7]. Initial infection by SARS-CoV-2 begins the incubation period (during which infectivity may peak), and continues until the onset of symptom presentation [8]. Beginning at the point of initial infection, and continuing through the incubation period and infectious course, SARS-CoV-2 binds to the angiotensin-converting enzyme 2 (ACE2) receptor on human lung epithelium. As described in the following, ACE2 is found on a variety of other cell types, including the vascular endothelium and intestinal epithelium (see Table 1). The widespread expression patterns of this receptor may contribute to the varied, systemic symptoms observed. Additionally, infection by SARS-CoV-2 may result in the disease state of COVID-19. The pulmonary phase (phase II) of COVID-19 is associated with an established pulmonary disease, caused by infection of local epithelium and viral multiplication. This stage is commonly associated with viral pneumonia, cough, and fever. Stage II may be further subdivided into stages IIa (pulmonary disease without hypoxia) and IIb (pulmonary disease with hypoxia) [9]. The hyperinflammation phase is when additional systemic, inflammatory symptoms coincide with SARS-CoV-2 infection and antiviral type I and III interferon production [10]. This phase of the disease aligns with the idea that COVID-19 is associated with severe endothelial cell infection and inflammation leading to an increase in cardiovascular complications. It is important to note that disease severity may be highly dependent on patient characteristics, including incidences of comorbidity (particularly of inflammatory conditions such as cancer and diabetes) [11].

### 2.1. Cytokine Storms and the Inflammatory Response

A “cytokine storm” is defined as an excessive immune response towards an external stimulus. Cytokines play an essential role in the innate immune system against viral infections. However, an excessive first-line response to viruses can sometimes become more harmful than beneficial [12]. Cytokine storms progress rapidly in a very complex manner, resulting in a high mortality rate. Huang et al. [13] showed a history of increased proinflammatory cytokines in SARS-CoV-2-related viruses (including SARS and Middle East Respiratory Syndrome-CoV (MERS-CoV)), with these patients displaying increases in IL-1β, IL-6, IL-12, IFNγ, IP10, and MCP1 similar to those found in MAS. Similarly, the study reported that COVID-19 patients also had an increase in proinflammatory cytokines, leading to the activation of T helper 1 cell responses. However, these cytokines led to a further increase in additional cytokines (GCSF, IP10, MCP1, MIP1A, and TNFα), which were associated with increased ICU admission and disease severity. Essentially, the release of copious amounts of pro-inflammatory cytokines induces and sustains the systemic inflammatory response in severe COVID-19. Following excessive cytokine release, SARS-CoV-2 infections lead to the activation of additional immune cells that harm healthy cells and disturb normal physiological functions [6,12]. For example, the release of interferons I and III, though antiviral in function, may cause significant damage to the lung epithelium following extended exposure, as may be observed in the cytokine storm. This tissue damage may worsen clinical outcomes [10].

### 2.2. Neutrophil Extracellular Traps (NETs)

An increased neutrophil to lymphocyte ratio, serum levels of inflammatory cytokines and chemokines, and inflammatory markers in blood have been associated with increased disease severity and death. However, the observed neutrophil insurgence is likely not an effective defense against viral pathogens and could be suppressing T cell-mediated antiviral activities [14]. In addition, increased inflammatory markers in blood of COVID-19 patients (including C-reactive protein, ferritin, and D-dimers) have been reported, suggesting that there is hyperactivity of the coagulation system [5,15,16]. An elevated neutrophil count in COVID-19 patients suggests the pathogenic role of neutrophil extracellular traps (NETs), which have been found to contribute to thrombosis in other similar pandemic viruses H1N1, SARS-CoV, and MERS-CoV [5].

NETs function to disarm pathogens through the use of molecular complexes targeting DNA and promoting its destruction. Common proteins such as neutrophil elastase, cathepsin G, and specific histones are associated with NETs [17]. NETs are commonly understood as the immune system’s first line of defense towards infection, either by the secretion of antimicrobial substances or the engulfment of the pathogen. However, it has shown that an overactive NETosis state can lead to more harm than help to the host. Moreover, NETs are responsible for initiating thrombotic events in arteries, veins, and microvasculature by activating the contact pathway of coagulation via electrostatic interactions [18] and the intrinsic pathway by presenting tissue factor [19,20].

Serum samples in patients with COVID-19 display large amounts of NET remnants including MPO–DNA complex, citrullinated histone H3, and cell-free DNA, promoting venous thrombosis and inflammation of the vessel wall [5]. The serum of COVID-19 patients seems to have contents which stimulate NETosis; the exact contents are yet to be understood and encourages more research concerning NETs in COVID-19 infected individuals. When added to control neutrophils, COVID-19 sera are potent stimulators of NETosis [5]. The presence of NET remnants, and the activation of NETosis via COVID-19 sera, may suggest that patients suffer from a pro-NETosis stage. This stage may contribute to virally damaged epithelial cells, activated platelets, activated endothelial cells, and excessive release of inflammatory cytokines. Consequently, inhibitors of NETs are of interest as a potential treatment to reduce the severity of SARs-CoV-2 infection [21].

## 3. Biochemical Considerations of SARS-CoV-2

### 3.1. SARS-CoV-2 Host Cell Entry

Infection of blood vessel endothelium leading to systemic circulation of the virus is virtually unheard of in previous viral outbreaks. Neither taxonomic sister virus SARS, nor additional pandemic virus H1N1 (influenza A of the family *Orthomyxoviridae*, the cause of both the 1918 influenza and the 2009 swine flu pandemics), showed such endothelial disruption [5,22]. Nevertheless, SARS-CoV-2 exhibits an ACE-mediated mechanism of endothelial infection. The coronavirus spike protein attaches to ACE2 to enter the host cell [23]. This receptor is commonly found on epithelial type II cells, renal, intestinal, cardiac, and endothelial cells [15,16]. As analyzed by Varga et al., the distribution of this receptor is largely consistent with the clinical findings for target organ failure (e.g., cardiac and renal) but does not fully correlate with the broad symptomatology [23]. This study found the presence of viral bodies and host inflammatory cells in several organs, suggesting that SARS-CoV-2 leads to the induction of endotheliitis [23]. Endothelial cell infection that proceeds via ACE2 shows how SARS-CoV-2 can replicate into a wide range of cells, which may explain some of the clinical symptoms found in COVID-19 patients. However, how tissues with restricted serum, and thereby viral particle, access become infected is unknown.

### 3.2. Viral Replication Cycle

Following the entry of SARS-CoV-2 into the host cell, host ribosomes bind to and facilitate the translation of the positive-sense viral RNA genome. Translation of the viral genome results in the production of polyproteins capable of forming the Replication–Transcription Complex subsequent to processing by viral protease activity. The Replication–Transcription Complex then acts to produce positive-sense genomic RNA for future packaging, as well as mRNA transcripts for viral structural and accessory proteins (nucleocapsid, envelope, membrane, and spike proteins). These viral mRNA transcripts are then trafficked to, and translated by, the endoplasmic reticulum of the host cell. The newly produced viral proteins, as well as the genomic RNA, are then packaged by the Golgi apparatus and exocytosed as a fully functional viral particle [24,25].

### 3.3. ACE2 Tropism of SARS-CoV-2

SARS-CoV-2 has been demonstrated to use the ACE2 receptor on human endothelial cells for cell entry. In fact, it has been shown that only coronaviruses which express the SARS-CoV spike protein can utilize the ACE2 receptor [26]. This was originally reported in a 2003 study conducted using the SARS-CoV permissive African green monkey kidney cell line Vero E6. Vero E6 have been subsequently used extensively to characterize this family of viruses and the molecular features of SARS-CoV spike protein–ACE2 receptor interaction [29,30,31].

SARS-CoV and SARS-CoV-2 both utilize a conserved spike protein (also called the S protein). In SARS-CoV, the S protein has two distinct subunits—S1 and S2. S1 was determined to have a high-affinity association with respective receptors in particular the ACE2 receptor [27,29]. Using a carboxy terminally tagged form of the S1 domain as bait, Vero E6 cell lysate was incubated and S1 immunoprecipitated. After gel purification, interacting proteins were identified by mass spectrometry. Three proteins were identified as putative S1 interactors with ACE2 (8 fragments which comprised 17% of human ACE2) being the strongest cell surface receptor candidate. This finding piqued the interest of researchers studying SARS as the tissue distribution and localization of ACE2 make it an appropriate receptor for SARS-CoV [30]. This work was then validated in a Vero E6 cell line cloned from human-lung endothelial cells where ACE2 -S1 binding was confirmed using blocking antibodies [31].

In a separate study, Transmembrane Serine Protease 2 (TMPRSS2) was shown to activate the viral S protein which affects viral entry but not replication [32]. TMPRSS2 accomplishes this by cleaving the glycoprotein hemaglutenin which is necessary for the fusion of viral and host cell membranes. This forces a conformational change after endocytosis and allows for viral invasion into the host cell cytoplasm [33].

Betacoronaviruses (such as SARS-CoV, SARS-CoV-2, and MERS) have only two distinct methods of entering endothelial cells. The first is through the endosomal pathway. It has been shown that MERS, which binds to and enters cells in a similar method (though with the utilization of the dipeptidyl peptidase-4 receptor) as SARS, can bind to cathepsin L in the endosome in the absence of TMPRSS2 and facilitate entry into the host cell cytoplasm [34]. However, this method results in a greater than 100-fold reduction in infectivity compared with TMPRSS2-mediated entry [32]. Notably, TMPRSS2 and ACE2 are found in their highest concentrations in endothelial cells located in the kidneys, as well as the lung and heart, where SARS and MERS coronaviruses produce the most life-threatening damage [1,23].

ACE2 contains two virus-binding locations which provide a tight bond between this protein and SARS-CoV-2 [35,36]. SARS-CoV-2 forms a larger binding surface with ACE2 than with SARS-CoV, increasing the binding affinity. Binding hotspots within ACE2 are designated as hotspot 31 and hotspot 353, which likely form weak salt bridges (electrostatic interaction between amino acid side chains at secondary and tertiary levels of organization) between amino acids in the native protein that are then broken upon binding of the virus spike protein [37]. Hotspot 31 breaks and forms two hydrogen bonds with the virus, whereas hotspot 353 breaks the weak salt bridge and forms a single hydrogen bond with the main chain of SARS-CoV-2 while maintaining one weak salt bridge. Both hotspots exhibit stronger and more stable binding through the SARS-CoV-2–ACE2 interface than the native ACE2 intramolecular interactions [35,36]. While ACE2 and TMPRSS2 are the most well-characterized viral entry factors, it is important to note that these proteins are not the only ones that have been reported to facilitate this process [38].

### 3.4. SARS-CoV-2 Spike Protein Molecular Mimicry: ENaC-Alpha, ACE2, and PCSK

SARS-CoV-2 portrays an evolutionary strategy that distinguishes it from other zoonotic origin strains, illustrating the possibility of human epithelial sodium channel alpha subunit (ENaC-alpha) mimicry in COVID-19. ENaC is associated with the renin–angiotensin–aldosterone system (RAAS) and regulates sodium ion concentration and water homeostasis in distal lung airway tissue [39]. Contrary to other areas of the human body, ENaC sodium transport is not involved in direct salt balance, but rather in maintaining appropriate hydration of the inner surface epithelial layer [39]. Therefore, the key role of ENaC in alveolar tissue is to control the volume of airway-surface liquids. In a study performed by Stoner et al., ENaC was evaluated in flat epithelia from toad urinary bladder and frog skin. The study portrayed the great specificity of ENaC for sodium; however, little was known about the exact mechanism of activation [40]. Importantly, ENaC needs to be proteolytically activated to function in controlling fluid reabsorption in the airways [40]. It is now reported that furin cleaves ENaC between arginine and serine residues in the 4th and 5th position (RSAR/SASS) to become activated [41].

When compared to SARS-CoV strains, a unique sequence insertion at the S1/S2 site of the spike protein is found in SARS-CoV-2 [42]. SARS-CoV-2 is coated with spike glycoprotein (S) that requires proteolytic cleavage for activation [41]. After interaction of S with the ACE2 receptor, SARS-CoV-2 entry into host cells occurs in two steps. Once the S1 subunit of the S protein engages the ACE2 receptor of the host cell, viral entry into the host cell is facilitated via S1/S2 cleavage [42]. The S2 site is then cleaved to allow the viral–host cell membranes to fuse [42]. The ability of the virus to facilitate the use of host mechanisms to cleave at S1/S2 furthers belief that SARS-CoV-2 has developed an evolutionarily beneficial insertion to achieve increased host infection. Walls et al. show that the furin-like cleavage motifs at the S1/S2 site are present in other viruses, but the exact mimicry of ENaC-alpha is unique to SARS-CoV-2 [42].

As explained earlier, the ACE2 receptors are present in a variety of different locations ranging from alveolar to intestinal cells [23]. Remarkably, ENaC-alpha are present in apical membranes of similar cells as those of ACE2 [23,41,43] leading to the hypothesis that SARS-CoV-2 leverages the protease network that is responsible for the cleavage of ENaC, which could subsequently lead to excessive sodium and liquid buildup in the lungs explaining clinical findings of ARDS. Anand et al. found that the viral receptor ACE2, ENaC-alpha, and furin were coexpressed in the colon (immature enterocytes) and pancreas (ductal cells) of human tissue and tongue (keratinocytes) of mouse cells [41]. Moreover, PCSK5 and PCSK7 are expressed in the intestinal, pancreas, nasal cavity, tracheal epithelial cells, and podocytes of the kidney, similarly to that of the ACE2 receptor [41].

## 4. Therapeutic Possibilities

Thus far, we have discussed the viral mechanisms of SARS-CoV-2 and resultant COVID-19 sequelae as they relate to endotheliitis and endothelial cell infection mediated by viral spike protein–ACE2 interaction. We now turn our focus towards potential therapies that can be derived from an understanding of infection and disease progression as fundamentally concerned with endothelial cells and their subsequent pathophysiology. When considering potential treatments for viral infections of this nature, targets may vary based upon treatment intention. The following discussion will concern both the prevention of cellular infection and symptomatic amelioration following infection.

### 4.1. Prevention of Cellular Infection

Prevention of initial cellular entry and infection should be considered separately from ameliorative therapies, as it may negate the need for further symptomatic treatment. As discussed previously, SARS-CoV-2 finds its receptor in the highly expressed ACE2 [44,45]. This transmembrane receptor functions primarily in the RAAS, as part of the body’s innate homeostatic balance, acting to maintain fluid balance and blood pressure [44,46]. An understanding of this initial viral binding event allows for the proposition of recombinant ACE2 as a competitor against viral binding to the endogenous receptor. If successful, recombinant soluble receptor domains serve to “distract” the virus from endogenous ACE2, thereby preventing or reducing cellular infection [44]. This approach has seen initial success in an April 2020 study of endothelial organoids. Using an anatomically and physiologically analogous endothelial tissue model, Monteil et al. was able to show a significant decrease (4–5-fold decrease) in viral RNA upon treatment with human recombinant soluble ACE2 (hrsACE2) at doses of 50–100 µg/mL [44]. The validity of this approach is bolstered by the recent successful completion of phase I and II safety trials of clinical grade hrsACE2 (GSK2586881) in the treatment of ARDS. It is important to note that the trial of GSK2586881 concerned itself with the ability of recombinant ACE2 to attenuate lung injury through its innate physiological actions, and not as a competitor for viral binding [44,47]. However, the safety of the drug as demonstrated in phase II trials, considered in conjunction with the proven ability to reduce viral load in an endothelial model, points to potential applications in this additional therapeutic role.

The viral spike protein itself may also present a therapeutic target. Published in 2020, in response to the current pandemic, a study conducted by Wang et al. has confirmed the first known human monoclonal antibody against SARS-CoV-2. Human monoclonal antibody 47D11 is a neutralizing antibody against the S1 subunit of the spike protein, and has been shown effective in the inhibition of cellular infection of VeroE6 cells in vitro. Additionally, as 47D11 shows cross-reactivity and neutralization of the S1 in both SARS-CoV and SARS-CoV-2, Wang et al. propose that this antibody may be effective in the treatment of future coronavirus strains. It is important to note that the specific mechanism of action of 47D11 remains unknown, and is thought to be unrelated to receptor-binding interference, which serves to further differentiate 47D11 from other similar potential therapeutics [48].

In addition to prevention of initial ACE2 binding, our previous discussion of SARS-CoV-2 activation allows for investigation into additional targets. Of these targets, the most prescient appears to be the TMPRSS2 [45,49]. As of June 2020, TMPRSS2 inhibitor camostat mesylate is undergoing phase II trials [50]. This is a placebo-controlled, double-blinded study, with an intervention group of 200 mg oral dosing three times daily for seven days in patients over 18 years of age [50]. The primary outcome measure of this study is viral load as assessed by qPCR from days 0 to 4. Secondary measures include changes in viral load at different time points after treatment, changes in symptom severity as assessed by the COVID-19 daily self-score tool, changes in infection status, body temperature, and symptom frequency [50]. Investigation into camostat mesylate for COVID-19 is based upon previous studies showing the efficacy of the drug candidate in SARS-CoV-1 [49] These studies were primarily interested in the epithelial cells of the lungs, and not the vasculature [49]. However, as direct endothelial infection relies upon the action of this serine protease similarly to epithelial cell infection, it remains possible that camostat mesylate may be efficacious in the prevention of endothelial cell infection. However, additional research into the potential effects on the vasculature is needed.

Another successful strategy to inhibit SARS-CoV-2 has been through the use of Remdesivir [51]. This drug has an adenosine nucleoside triphosphate analog that interferes with viral RNA polymerase and is classified as a delayed chain terminator [52]. Remdesivir has exhibited promise in the clinic in early studies resulting in a median recovery time of 11 days versus 15 days for placebo [51]. In addition, mortality was reduced from 11.9% to 7.1% by Remdesivir [51]. This drug has shown promising results. However, the morbidity and mortality rate is still cause for concern. Therapeutic approaches concerned with the abruption of viral replication processes (including Remdesivir) may present relevant targets for future research not reliant on ACE2 and TMPRSS2. These targets may include those relating to transcription, metabolism, and biomolecule synthesis [53].

### 4.2. Symptom Amelioration

Endothelial pathology as a result of COVID-19 progression can be roughly broken into three subcategories: coagulopathy, inflammation (including cytokine storm), and edema. As such, potential endothelial concerned treatments for COVID-19 may be subdivided similarly. Though both coagulative and edemic pathologies have their roots in inflammation, there are additional considerations that warrant the division as follows.

#### 4.2.1. Coagulation

A primary vascular concern of COVID-19 appears to be that of rampantly dysregulated thrombotic events [54,55]. These clotting abnormalities have resulted in stroke and disseminated intravascular coagulation (DIC) in severe COVID-19 patients, and may represent a significant cause of mortality [54,56]. Though blood thinners, such as heparin, have been shown to reduce mortality in severe COVID-19 cases, this acts only after the development of the clotting pathology, and not to prevent the dysregulation of the clotting cascade [54,55]. To prevent aberrant thrombosis, it is helpful to investigate the endothelial pathology that allows for upregulation of the clotting cascade. In COVID-19, SARS-CoV-2 infection of endothelial cells and subsequent cell death, allows for pathological exposure of blood to the thrombogenic basement membrane of the blood vessel which contains collagen [57,58,59]. Collagen exposure activates the clotting cascade. As such, actors within the clotting cascade present potential therapeutic targets. Of these targets, P-selectin may be appropriate, as antibodies to this cell adhesion molecule have been used to prevent thrombosis [58]. Additionally, pro-inflammatory cytokines interleukin 1 beta (IL-1β) and tumor necrosis factor (TNF), are integral to the clotting cascade as they function cyclically to trigger platelet production and the upregulation of additional adhesion molecules [56,60]. As such, antibodies against these cytokines may help to temper the hypercoagulative state and prevent fatal DIC. As this clotting pathology is often inflammatory in nature, many of the proposed therapies in the treatment of the cytokine storm may be efficacious in the treatment of the clotting abnormalities and will be discussed in more detail in the following section.

#### 4.2.2. Inflammation as a Treatable Pathology

A cytokine storm refers to a hyperinflammatory condition related to the uncontrolled release of cytokines [12]. This condition is seen as controlled inflammation (pain, heat, redness, and swelling) that occurs as a healing response to injury and infection, becomes overactive and systemic as opposed to strictly regulated and locally active [61]. In COVID-19, the cytokine storm is seen to worsen the inflammatory symptoms of the disease, including the aforementioned clotting pathologies and subsequent multi-system organ damage [59,62]. Therapeutic candidates to treat this inflammatory condition include corticosteroids and monoclonal antibodies, among others [59,63]. An ongoing trial, currently in phase III, tests the effects of cytokine-targeting medications in individual and combination therapy [63]. This study tests IL-6 antibodies Tocilizumab (anti IL-6R) and Siltuximab (anti IL-6), as well as IL-1β receptor antagonist Anakinra. Intervention groups include individual treatment with each of the three pharmaceuticals, as well as combination therapy with Anakinra and either Tocilizumab or Siltuximab. The primary outcome measure of this trial is time to clinical improvement as assessed by hospital discharge or improvement in clinical indicators. Secondary outcome measures include, among others, incidences of adverse events, mean change in oxygenation, and mean change in clinical scores [63]. The successful use of anti-interleukin drugs to treat the inflammatory symptoms seen in severe COVID-19 would have marked effects on endothelial pathology as these cells are highly responsive to cytokine signaling [59]. This ameliorative effect would be seen in the downregulation of the prothrombotic cascades as initiated by cytokine signaling, as well as decreased inflammatory leukocytic cascades.

As mentioned, corticosteroids may be effective in the treatment of inflammatory pathology. A randomized controlled trial conducted by the RECOVERY Collaborative Group tests the efficacy of dexamethasone in the treatment of hospitalized COVID-19 patients. The primary outcome measure of this study is mortality at 28 days. Patients received oral or intravenous dosing at 6 mg/day or continued care without corticosteroid intervention. This study found that dexamethasone was effective in lowering mortality at 28 days when compared to control patients receiving mechanical ventilation or supplemental oxygen. However, the mortality rate among patients included in the intervention group was not found to be lower when compared to hospitalized patients not receiving ventilation or external oxygen [64].

#### 4.2.3. Edema

Edema, or swelling, is a fundamental inflammatory response that occurs primarily due to vascular leakage. Similar to other hallmark inflammatory responses of pain, redness, and heat; edema occurs primarily as a result of endothelial cell function or dysfunction [61]. As reviewed by Teuwin et al., in COVID-19, the overactive edemic response may occur due to a weakening of the endothelium as a result of cell death secondary to direct endothelial infection [59]. Additionally, the actions of cytokines IL-1β and TNF weaken intercellular tight junctions and result in the overproduction of hyaluronic acid (HA) in the extracellular matrix of endothelial cells [59].

Increases in vascular leakage due to cell death following direct endothelial infection may be lessened with the use of antiviral medications. These pharmaceuticals may prevent intracellular viral replication and resultant cell death [59,65]. A potential agent in this approach is Ribavirin, which may inhibit viral RNA polymerase and subsequent viral replication. Ribavirin was tested in both SARS and MERS and found significant safety concerns limiting its efficacy, though as of now, suffers from a dearth of SARS-CoV-2 research [65,66,67]. Additionally, previous clinical trials showing toxicity related to necessary dosage were concerned mainly with the complete cessation of viral activity, and not with symptomatic amelioration. Lower dosages that were shown to be ineffective when used as the sole antiviral treatment in SARS, may be effective in combination therapy or in purely symptomatic aims [65,66,67]. The weakening of the endothelium may also occur as a result of increased contractility and the loosening of intercellular tight junctions due to the inflammatory properties of cytokines [59,68]. Again, edema may be treated similarly to cytokine storm pathology as the causative agents of inflammation are cytokines such as IL-6 [11].

As mentioned, edema may be inflammatory in origin. In COVID-19, the actions of cytokines IL-1β and TNF can be seen to upregulate HA-synthase-2, resulting in increased HA concentrations in the extracellular matrix of endothelial cells. This leads to marked water retention and vascular leakage and may therefore contribute to severe respiratory pathology and ARDS [59,69]. Potential therapeutics in this aim include anti-inflammatory drugs against IL-1β as discussed previously.

## 5. Extravascular Effects of Endothelial Infection

Through this review, we have discussed the mechanisms, pathologies, and proposed treatments of SARS-CoV-2 infection with a focus on the effects of direct endothelial infection and dysfunction. Though the vasculature itself appears to play a large role in the pathogenesis of the disease, there is additional merit in a discussion of non-vascular infection and pathology that occurs as a function of initial endothelial infection. The following will discuss potential endothelial-derived mechanisms of extravascular infection and pathology.

The healthy endothelium has roles beyond those of a vascular nature. In uninfected cells, these functions allow for and maintain the health of endothelial tissue, as well as the overall health of the organism [70,71]. However, upon endothelial infection, innate processes may become factors driving further pathogenesis. The transdifferentiative capacity of endothelial cells, as well as the innate exocytotic machinery of these cells, may be such infectious considerations [71,72,73].

Transdifferentiation describes the process whereby a mature cell undergoes transformation into another cell type [70,74]. Endothelial cells retain this ability into maturity and have been found to assume multiple extravascular phenotypes, as shown by fluorescent fate mapping [72,74]. Evidence suggests that descendent cell types may include cerebellar granule neurons, pancreatic acinar and β cells, duodenal and ileal crypt cells, ependymal cells, and skeletal myocytes among others [74]. Additionally, genetic fate mapping in rhabdomyosarcoma has given further credence to the transdifferentiative role of endothelial progenitors in disease pathogenesis [72]. This propensity for transdifferentiation does not, on its own, imply a further infectious route for SARS-CoV-2 from endothelium to descendent cell. However, when considered in conjunction with the extravascular labeling that results from intravenous Adeno-associated virus injection (a common labeling method in model systems), relevant conclusions may be drawn. Notably, if the ability of intravenous Adenovirus to label extravascular cells is due to endothelial transdifferentiation, then this labeling (Adenovirus infection in descendant cells upon transdifferentiation) may allow for the inference of a similar route for SARS-CoV-2 infection [73,74,75]. In other words, the transdifferentiation of endothelial cells infected with SARS-CoV-2 may result in the creation of extravascular descendent cells that are similarly infected (much in the same way that Adenovirus injection results in extravascular labeling), thereby furthering systemic SARS-CoV-2 infection. This analysis should not be interpreted to imply significant similarity between SARS-CoV-2 and Adenovirus beyond that as a model of endothelial infection and viral propagation subsequent to transdifferentiation. The final consideration of endothelial transdifferentiation is that of process and function. Due to the limited conditions under which transdifferentiation successfully occurs, endothelial SARS-CoV-2 infection may alter or disrupt this process [70]. Alternatively, successful transdifferentiation of an infected cell may result in a descendent cell with pathologies independent from those occurring due to an active infection, though more research is needed.

In addition to transdifferentiation, exocytosis is a possible route for endothelial-derived extravascular SARS-CoV-2 infection. Similar to transdifferentiation, exocytosis can be considered as an innate function of healthy endothelial cells, which may become a driver of further infection and pathology following viral infection with SARS-CoV-2 [71,73,76]. As reviewed by Hromada et al., non-pathological endothelial extracellular vesicles have functions relating to immunity, cell-to-cell communication, and tissue regeneration [71]. As discussed, SARS-CoV-2 is a membrane-bound virus that binds to ACE2 [44,45]. Viral binding and enzymatic activities support viral membrane fusion with the endothelial cell membrane, thereby allowing viral RNAs, transcription factors, and additional viral proteins to enter the endothelial cell and begin the viral replicative processes that signify an active infection [45]. Exocytotic mechanisms may persist during a viral infection, and thus contribute to pathogenesis. In this case, a portion of the exocytosed cell membrane in any vesicle may be of viral origin, and thus may contain the spike proteins needed for viral binding [73,77]. Additionally, during exocytosis of the extracellular vesicle, intracellular endothelial cell contents may be packaged within the vesicle. The inclusion of these components within an exocytosed vesicle is a non-pathogenic mechanism that allows for cell-to-cell communication and transport [76]. However, in the case of an active SARS-CoV-2 infection, viral components within the endothelial cell may also be packaged within the vesicle [76]. As a result, there is potential for the creation of an endothelial extracellular vesicle possessing membrane-bound spike proteins externally, as well as viral RNA and transcription factors internally [73,76]. This may allow for an endothelial extracellular vesicle to effectively become a virus-like particle capable of transmitting SARS-CoV-2 to other vascular and extravascular cells. Additionally, uninfected endothelial cells may play a role in increasing susceptibility to SARS-CoV-2 infection. This increased susceptibility may occur due to the exocytosis and subsequent fusion of endothelial vesicles possessing ACE2 within their membrane [73,77]. Conferred SARS-CoV-2 susceptibility may subject the recipient cells to the viral pathology upon infection.

## 6. Conclusions

Combating the COVID-19 pandemic will require a comprehensive understanding of the SARS-CoV-2 pathology caused at the molecular and cellular levels. Given the high vaccine failure rate for many common seasonal viruses, researchers should continue to explore both vaccine approaches, and additional antiviral strategies. In this review, we have focused our attention on endothelial cells as an emerging COVID-19 contributor and potential therapeutic target. This review has covered the clinical COVID-19 manifestations that could be linked to endothelial cell dysfunction, the molecular biology of SARS-CoV-2 entry into ACE2-expressing endothelial cells, immune reactions that are both dictated by and perpetuated upon endothelial cells, current COVID-19 therapies reported to address aspects of endothelial cell biology, and perturbation of canonical and non-canonical endothelial cell biological processes (exosome production and transdifferentiation) by this unique virus. Therefore, harnessing endothelial cell biology to prevent infection, reduce inflammatory effects and avoid SARS-CoV-2-mediated disturbances of natural endothelial cell physiology could provide important avenues for improving patient outcomes.

## Figures and Tables

**Table 1:** Cell type and tissue distribution of ACE2 and Transmembrane Serine Protease 2 (TMPRSS2) protein expression. Detection of ACE2 or TMPRSS2 is indicated by a plus (+) and unsuccessful detection is also shown (-).

Cell Type	Tissue	ACE2 Expression	TMPRSS2 Expression	Reference
Goblet/Basal/Ciliated Cells	Nasal Cavity	-	+	[17,18]
Secretory/Basal/Multiciliated	Lung	+	+	[19]
Epithelial Enterocytes	Ileum	+	+	[21]
Endothelial Cells	Vasculature	+	+	[15]
Myocyte Cells	Heart	+	-	[22]
Pericyte Cells	Heart	+	-	[22]
Corneal Epithelium	Eye	+	+	[23]
Cholanglocytes	Liver	+	-	[24]
Epithelial Cells	Bladder	-	+	[25]
Fibroblast Cells	Bladder	-	+	[25]
Proximal Tubule	Kidney	+	+	[26]
Ductal Epithelium	Pancreas	+	+	[27]
Epithelial Cells	Prostate/Testis	-	+	[28]
